# Artificial intelligence in wearable seizure detection devices: current technologies and future directions

**DOI:** 10.3389/fneur.2026.1756895

**Published:** 2026-03-09

**Authors:** Tiffany Jiaqi Ho, Bridget Elaine LaMonica Ostrem, James Michael Hillis

**Affiliations:** 1 Department of Molecular and Cell Biology, University of California, Berkeley, Berkeley, CA, United States; 2 Department of Neurology, University of California, San Francisco, San Francisco, CA, United States; 3 Department of Neurology, Massachusetts General Hospital, Boston, MA, United States; 4 Mass General Brigham AI, Mass General Brigham, Boston, MA, United States; 5 Harvard Medical School, Boston, MA, United States

**Keywords:** artificial intelligence, epilepsy, machine learning, seizure, wearable

## Abstract

Epilepsy affects millions of people worldwide, driving the need for advanced methods to monitor patients’ health and seizure activity. Recent advances in wearable technologies have enabled continuous collection of physiological data to support real-time seizure detection in the real-world. This review presents a targeted synthesis of 23 studies evaluating wearable devices and their associated artificial intelligence (AI) algorithms for automated seizure detection. Both wrist- and ear-based systems demonstrate high sensitivity, with performance influenced by device design, signal reliability, and analytic approach. The main challenges include reducing false alarms and maintaining data integrity during everyday use. More recent studies highlight the ability to anticipate seizures before they occur, marking a promising step toward improving safety and well-being for people living with epilepsy. Ongoing efforts to identify reliable physiological markers and to evaluate device performance across diverse populations are key to integrating wearable technologies for seizure detection into routine medical care.

## 1 Introduction

Epilepsy affects over 3 million adults and children in the United States, with the Epilepsy Foundation reporting that 1 in 26 people in the United States are diagnosed with epilepsy in their lifetime (1). Seizures, the defining feature of epilepsy, arise from abnormal bursts of electrical activity in the brain. Seizures are broadly classified as focal or generalized with manifestations including limb stiffening, rhythmic jerking and impaired awareness (2). Treatment of epilepsy is focused on preventing seizures through the use of antiseizure medications, surgical resection, and implantable devices, among other approaches (3–5).

The gold-standard diagnostic test for seizure detection is electroencephalography (EEG), which evaluates electrical activity in the brain through the use of scalp or implanted electrodes. EEG testing is often performed in the outpatient setting, with typical duration of less than an hour. Inpatient long-term EEG or home ambulatory EEG monitoring can last for many days to over a week. The interpretation of EEG may benefit from automated methods including machine learning techniques (6–8). The biggest challenge with EEG use is that it can only provide diagnostic information while the EEG electrodes are in place. In contrast, wearable seizure detection devices provide an opportunity for accessible and continuous monitoring (9–11). They also offer advantages in cost, convenience and comfort compared with EEG, and can facilitate communication between people living with epilepsy, their caregivers and their clinicians through automated alerts. Similar to EEG interpretation, these wearable devices often leverage machine learning techniques to interpret the vast quantities of data that they produce.

This review synthesizes the existing literature regarding wearable devices that detect or predict seizures. We specifically aim to examine devices that compare biosignals to reference data obtained from EEG. We also cover signal acquisition techniques from EEG and other wearable devices, emerging technologies including wristbands and earpieces, and the application of AI to these areas.

## 2 Methods

**Figure 1:**
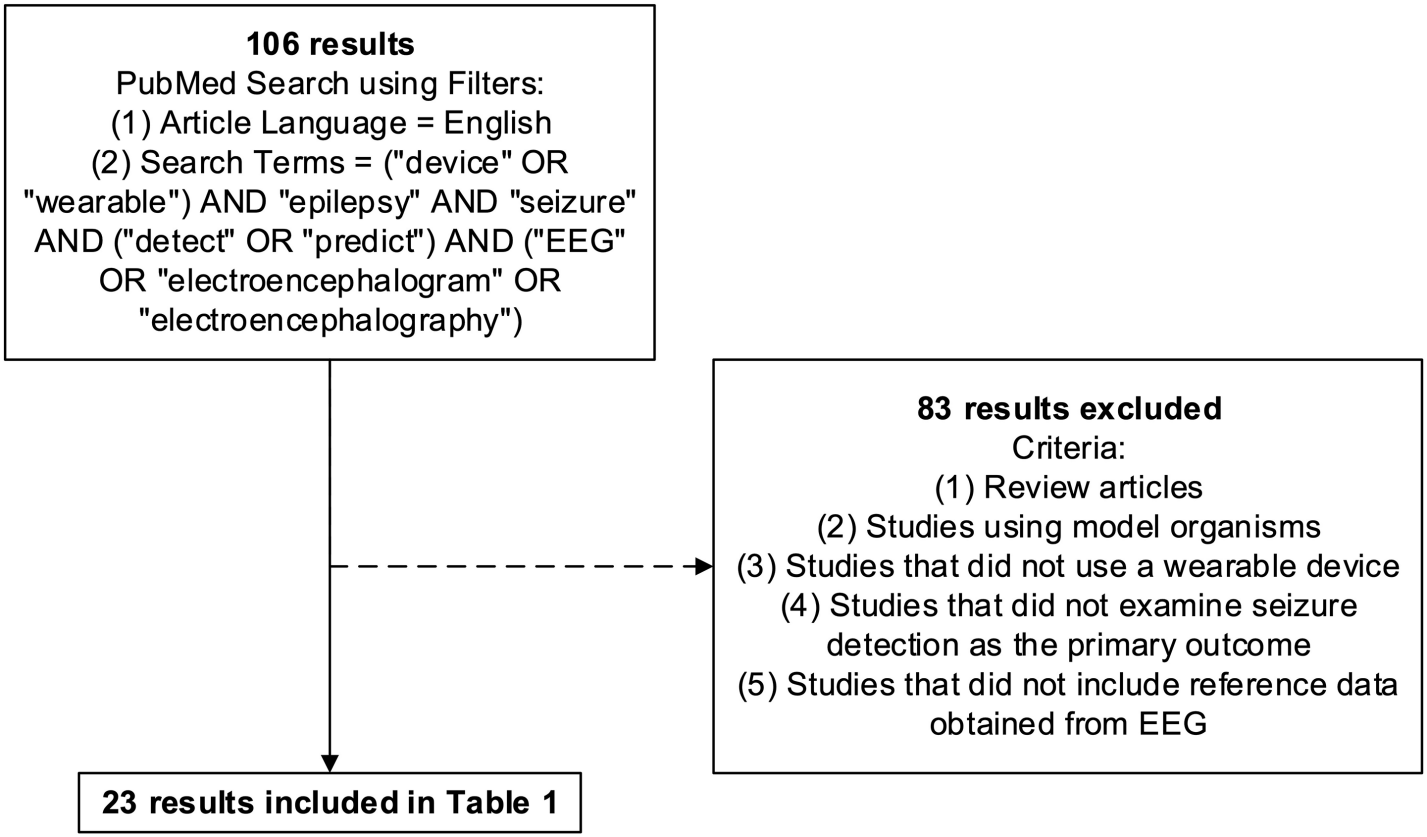
Flowchart showing the identification and screening of studies from a focused PubMed search.

We identified relevant studies through a focused PubMed search conducted on August 29, 2025, followed by manual screening according to the inclusion criteria outlined in Figure 1. Only articles published in English were included. Search terms included “device” or “wearable,” “epilepsy,” “seizure,” “detect” or “predict,” and “EEG” or “electroencephalogram” or “electroencephalography,” and were designed to capture original research on wearable technologies capable of detecting or predicting epileptic seizures, with EEG used as a reference standard. Additional studies were considered for the broader discussion in this review and were identified through manual reference screening.

## 3 EEG and biosignals for seizure detection

Seizure detection requires identification of relevant changes in physiological signals, also called biosignals. Among the diagnostic tools, EEG provides the most direct window into brain activity. However, other biosignals, including heart rate, electrodermal activity (EDA), and body temperature may show measurable changes during seizures. While each signal in isolation may offer only a limited perspective, they can provide a more reliable picture of a patient’s situation when integrated together.

### 3.1 EEG

EEG uses electrodes placed on or near the brain to measure electrical activity. Intracranial EEG provides the highest spatial resolution, allowing precise localization of signals to specific brain regions (12). However, this method requires invasive neurosurgery and carries significant risks. Scalp EEG involves placing electrodes on the scalp that typically follow the International 10–20 system (13). This noninvasive method is widely used in clinical practice and, although less precise than intracranial EEG, provides greater insight into seizure-related neural activity than indirect physiological signals such as EDA. Video EEG combines scalp EEG with synchronized video recording to correlate electrical activity with observable movements and behaviors. In the context of long-term seizure detection, however, these EEG modalities are unsuitable for everyday use, as they rely on specialized equipment and the maintenance of scalp electrodes, which require frequent adjustments and can cause minor scalp injury over time.

Wearable EEG devices with a reduced number of electrodes have been developed to address some of the practical limitations of conventional EEG. Many of these EEG devices are worn around the ear, and involve the placement of small electrodes on the outer ear canal, on the concha of the ear, or on the mastoid bone behind the earlobes (14). Although signals recorded from these locations are often limited to the temporal lobes, these devices still capture EEG signals from the scalp and are more amenable to long-term use compared with traditional scalp EEG. Headband devices are less discreet but may allow for a larger number of electrodes (15). While a comprehensive overview of wearable EEG devices in development and clinical use is outside of the scope of this review, several recent publications address this topic (16, 17).

### 3.2 Biosignals

Research has increasingly explored biosignals outside of EEG for their accessibility and ease of integration into wearable devices. Among these signals, movement has been the most extensively studied, particularly in generalized tonic clonic seizures where rhythmic jerking movements of the extremities can be captured with motion sensors. Accelerometer and gyrometer sensors are commonly found in wrist-worn devices that track movement. Accelerometers measure linear acceleration, making them well-suited for detecting sudden jerking movements, while gyrometers capture angular velocity, allowing for recognition of more complex, repetitive motor patterns (18). Together these signals provide a detailed picture of bodily motions for devices to differentiate epileptic activity from normal daily movements.

While motion sensors capture external manifestations of seizures, internal physiological signals provide additional insight into the body’s systemic responses. EDA, also referred to as galvanic skin response, measures changes in the skin’s electrical resistance or conductance that are attributed to sweat gland activity. Studies have demonstrated changes in EDA in individuals with epilepsy compared to individuals without epilepsy, and specifically during epileptic seizures (19–22). Electrocardiography (ECG) and surface electromyography (sEMG) record electrical signals from the heart and skeletal muscle, respectively, that may also change during seizure activity (23). Respiratory inductance plethysmography compares changes in chest and abdominal movements to record breathing patterns. Photoplethysmography (PPG) tracks changes in blood volume using light absorption and is often used to estimate heart rate and blood volume pulse. Previous work has reported significant changes in PPG before and after seizures (24), supporting its potential as a seizure-related biosignal. Body temperature may change during a seizure as well (21, 22). While the particular signals that contribute to seizure detection vary from patient to patient, approaches integrating multiple signal types have shown the strongest performance (25).

### 3.3 Challenges in signal quality

Obtaining reliable data for seizure detection involves two central challenges: ensuring that captured signals are true signals and recording over extended periods of time in real-world settings. Signal quality is often confounded by artifacts: wearable recordings are particularly susceptible to motion artifacts, physiological noise, and data loss when patients remove or adjust the device (26). Seizures themselves can introduce abrupt movements or muscle activity that further distort the recorded signals (27). Ongoing efforts focus on improving signal-to-noise ratio through device engineering and data processing. Several studies report excluding participants due to device malfunctions or incomplete recordings, underscoring the limitations of current wearable designs. While historically patients needed to visit a hospital or clinic to record EEG and other biosignals, the technological advances of miniaturization and portability have shifted the focus to integrating multiple sensing modalities into smaller, more robust wearables.

## 4 Emerging technologies

Among wearables that capture physiological signals to monitor health, wristbands and earpieces have emerged as two promising device designs for seizure detection. These device types are prominent in the structured review detailed in Table 1.

**Table 1:** Key studies evaluating wearable devices for automated seizure detection.

Reference	*n*	Device(s)	Primary data	Reference data	AI	Method(s) for detection	Results
Heldberg et al. (40)	8	Empatica E3 wristband (Empatica Inc., Milan, Italy)	ACC; EDA	Video EEG	RF, kNN	RF classifier; kNN classifier	The RF classifier had 90.5% sensitivity, 5.6% precision, 93.3% specificity for motor seizures and 85.3% sensitivity, 12.3% precision, 96.8% specificity for nonmotor seizures. The kNN classifier had 76.2% sensitivity, 4.6% precision, 93.4% specificity for motor seizures and 97.1% sensitivity, 9.6% precision, 92.9% specificity for nonmotor seizures.
Joo et al. (66)	12	LSM303DLM 3-axis accelerometer and magnetometer (STMicroelectronics, Geneva, Switzerland) in a novel wristband unit	ACC	Video EEG	No AI	Spectral analysis via seizure correlation ratio (SCR); temporal analysis via standard deviation	The spectral analysis method had a sensitivity of 100% and 2.0 FPs/24 h. The temporal analysis method had a sensitivity of 90% and 11.8 FPs/24 h.
Vandecasteele et al. (67)	11	eMotion Faros 180° (Mega Electronics Ltd., Kuopio, Finland); Empatica E4 wristband (Empatica Inc., Milan, Italy)	Wearable ECG; hospital ECG; wearable PPG	Scalp EEG	SVM	Support vector machine (SVM) classifier with a Gaussian kernel	Hospital ECG had a sensitivity of 57%, 1.92 FPs/h, and PPV of 1.93%. Wearable ECG had a sensitivity of 70%, 2.11 FPs/h, and PPV of 2.15%. Wearable PPG had a sensitivity of 32%, 1.80 FPs/h, and PPV of 1.12%.
Zibrandtsen et al. (42)	15	4 recording electrodes embedded in each 3D printed custom earpiece	In-ear EEG	Scalp EEG	No AI	Two neurophysiologists evaluated both scalp EEG and ear EEG data	Neurophysiologist 1 had a sensitivity of 56% and a specificity of 1 for both scalp EEG and ear EEG. Neurophysiologist 2 had a sensitivity of 96% and a specificity of 0.90 for scalp EEG, and a specificity of 92% and a specificity of 0.87 for ear EEG.
Johansson et al. (68)	75	Novel sensor (RISE Acreo, Sweden); Shimmer3 (Shimmer Research, Ireland)	ACC	Video EEG	SVM, RF, kNN	kNN with 5 neighbors; SVM with a linear kernel; RF with 30 trees	kNN had a sensitivity of 100%, 0.05 FP/h, and 1.2 FP/24 h. SVM had a sensitivity of 90%, 0.02 FP/h, and 0.48 FP/24 h. RF had a sensitivity of 90%, 0.01 FP/h, and 0.24 FP/24 h.
Naganur et al. (69)	26	Apple iPod Touch, 4th generation (Apple Inc., Cupertino, CA, USA)	ACC	Video EEG	SVM	SVM with a Gaussian radial basis kernel	For psychogenic non-epileptic seizures (PNES), the algorithm had a sensitivity of 100%, PPV of 81.3%, and NPV of 100%. For epileptic seizures (ES), the algorithm had a sensitivity of 72.7%.
Mittlesteadt et al. (37)	12	Fitbit Charge 2 smart watch (Fitbit, San Francisco, CA, USA)	Heart rate	EEG	NN	Neural network model using a 3-layer multilayer perceptron (MLP)	The algorithm had a ROC AUC of 0.58 with a 95% confidence interval of [0.56, 0.60].
Vandecasteele et al. (44)	54	4 electrodes glued to the skin behind the ears (two on each side)	Behind-the-ear EEG	Video EEG	SVM	One neurologist visually evaluated behind-the-ear EEG data; SVM with a radial basis function kernel	The neurologist had an average sensitivity of 65.7% and specificity of 94.4%. The patient-independent (PI) model had a sensitivity of 72.7% and 34.2 FPs/24 h for video EEG. The PI model had a sensitivity of 64.1% and 2.8 FPs/24 h for behind-the-ear EEG. The patient-specific (PS) model had an average sensitivity of 63.4% and 0.88 FP/24 h for video EEG. The PS model had an average sensitivity of 69.1% and 0.49 FP/24 h for behind-the-ear EEG.
You et al. (45)	12	NicoletOne™ EEG System (Natus Medical Incorporated, WI, USA)	Behind-the-ear EEG	Intracranial EEG; video EEG	GAN	Generative adversarial network (GAN) from a deep convolutional generative adversarial network	The algorithm had a sensitivity of 96.6% and 0.14 FA/h.
Böttcher et al. (62)	10	Empatica E4 wristband (Empatica Inc., Milan, Italy)	ACC; EDA	Video EEG	GTBM	Gradient tree boosting machine (GTBM)	The training set had a sensitivity of 100% and 0.46 FP/24 h. The test set had a sensitivity of 91% without any FPs. An expanded test set had 0.37 FP/24 h.
Nasseri et al. (63)	38	Empatica E4 wristband (Empatica Inc., Milan, Italy)	ACC; BVP; EDA; TEMP; estimated HR	Intracranial EEG	NN	Three-layer LSTM recursive NN classifier	The algorithm had a mean AUC of 0.98, sensitivity of 93%, and 2.3 FAs/day.
Onorati et al. (70)	152	Empatica E4 wristband (Empatica Inc., Milan, Italy); Empatica Embrace wristband (Empatica Inc., Milan, Italy)	ACC; EDA	Video EEG	ML	Three neurologists evaluated video EEG data; machine learning detection algorithm	For the pediatric population, the algorithm had a sensitivity of 92% and 1.26 FAs/24 h. For the adult population, the algorithm had a sensitivity of 94% and 0.57 FA/24 h.
Swinnen et al., 2021 (46)	12	2 Ambu Neuroline cup electrodes (Ambu, Denmark) attached behind each ear connected to the Sensor Dot (Byteflies, Belgium) device	Behind-the-ear EEG	Video EEG	SVM	Five epileptologists evaluated behind-the-ear EEG data; SVM with a radial basis function kernel	The epileptologists had a median sensitivity of 81% and a median precision of 89%. The initial algorithm had a sensitivity of 99.67% and 2.39 FPs/h. The postprocessed algorithm had a sensitivity of 98.3% and 0.91 FP/h.
Tang et al. (71)	94	Empatica E4 wristband (Empatica Inc., Milan, Italy)	TEMP; EDA; ACC; PPG to calculate BVP	Video EEG	CNN	CNN model with two convolutional layers	The seizure type-specific model had ROC AUCs greater than 0.6 for eight of nine different seizure types. The generalized model had ROC AUCs greater than 0.6 for all nine seizure types. For both models, combination of ACC and BVP data had the best average ROC AUCs.
You et al. (47)	16	NicoletOne™ EEG System (Natus Medical Incorporated, WI, USA)	Behind-the-ear EEG	Intracranial EEG; video EEG	NN	Two epileptologists evaluated intracranial EEG data; deep learning approach applying recurrent NN structures to variational autoencoder (VAE)	Vanilla RNN had a mean AUROC of 0.822, LSTM had a mean AUROC of 0.841, and GRU had a mean AUROC of 0.852. The anomaly detecting VAE algorithm had a mean AUROC of 0.8803 for the patients. The personalized anomaly detecting VAE algorithm had a mean AUROC of 0.908.
Proost et al. (72)	49	2 behind-the-ear adhesive Dry EEG electrodes (Byteflies, Belgium) or Hydrogel EEG electrodes (Byteflies, Belgium) or Ambu Neuroline surface electrodes (Ambu, Denmark) connected to the Sensor Dot (Byteflies, Belgium) device	sEMG; ECG; ACC	Video EEG	No AI	One EEG technologist scored and one childhood epileptologist validated video EEG data; a neurologist evaluated data from the wearable device	The full cohort had a sensitivity of 41%, PPV of 9%, and a median of 0.75 FA/h. Nighttime seizures ≥10s had a sensitivity of 66% and PPV of 66%. Nighttime seizures ≥20s had a sensitivity of 62% and PPV of 82%.
Yu et al. (73)	166	Empatica E4 wristband (Empatica Inc., Milan, Italy)	TEMP; EDA; ACC; BVP (from PPG)	Video EEG	CNN	CNN benchmark model with two convolutional layers; LSTM network building on one-dimensional convolutional filters	For the CNN model, combination of ACC and BVP data had the best performance with a sensitivity of 77.2%, FPR of 31.7%, and detection delay of 25.9 s. For the CNN-LSTM model, ACC + BVP had an AUC-ROC of 0.789, sensitivity of 83.9%, and FPR of 35.3%.
Gharbi et al. (50)	42	Hexoskin connected shirt (Carré Technologies Inc., Quebec, Canada)	ECG; ACC; two-channel RIP	Video EEG	ML	Three epileptologists evaluated video EEG data; XGBoost ML algorithm	The XGBoost algorithm on data extracted using 15 s epochs had a sensitivity of 84.8% and 0.55 FA/24 h. Epoch size of 10s had a sensitivity of 81.8% and 0.52 FA/24 h. Epoch size of 20s had a sensitivity of 78.8% and 0.49 FA/24 h. Epoch size of 25 s had a sensitivity of 83.3% and 0.51 FA/24 h.
Joyner et al. (43)	20	2 electrodes were placed in the cymba conchae and in the canal of each ear within earbuds customized by shape to each patient’s ears	In-ear EEG	Intracranial EEG; scalp EEG	No AI	Two epileptologists evaluated scalp, intracranial, and in-ear EEG	In-ear EEG seizure detection had an average sensitivity of 87.5% with 6 false negatives and <0.1 FP/24 h. Intracranial EEG had an average sensitivity of 87.5% and 0.147 FP/24 h. Scalp EEG had an average sensitivity of 85% and 0.035 FP/24 h.
Larsen et al. (41)	18	Wrist-worn device with accelerometer and gyroscope (Danish Care Technology, Denmark)	ACC; GYR	Video EEG; ECG; sEMG	NN	Artificial NN sequential model with layers including GaussianNoise, Dropout, Dense, and Activation layers	The test dataset had a sensitivity of 100, 95% CI of [0.69, 1.00], and 0.023 FA/h. The entire dataset had an average sensitivity of 95% and a 95% CI of [0.89, 0.99].
Vakilna et al. (39)	36	Samsung SM-R800 Watch (Samsung Electronics Co., Ltd., South Korea)	ACC; GYR; PPG; HR (from PPG)	Video EEG	RF	One epileptologist evaluated video EEG data; RF algorithm	In leave-one-patient-out cross-validation (LOPO CV) on generalized convulsive seizure (GCS) patients, the algorithm had a sensitivity of 0.87, 95% CI of [0.62, 0.96], and 0.21 FA/24 h. FAR testing in non-seizure patients resulted in 0.28 FA/24 h. In “fixed-and-frozen” prospective testing, the algorithm had a sensitivity of 100% and 0.25 FA/24 h.
Lehnen et al. (48)	32	2 silicone earpieces each with 2 electrodes in the BrainSD device (Department of Computer Science and Engineering, UT Arlington, Texas, USA)	Behind-the-ear EEG	Video EEG	SVM, RF, kNN	Two epileptologists evaluated video EEG data; SVM classifier; kNN classifier; RF classifier	SVM had an accuracy of 95.3%, sensitivity of 97%, and best overall performance of 96%. kNN had an accuracy of 92.8%. RF had an accuracy of 92.8%.
Newton et al. (74)	158	4 REMI sensors (Epitel, Inc., UT, USA) placed bilaterally between the F7-Fp1, F8-Fp2, T5-T3, and T6-T4 electrode locations	Scalp EEG (REMI)	Video EEG	ML	Three expert reviewers evaluated the video EEG data; XGBoost ML binary classifier	On the event level, the algorithm had a sensitivity of 86.2% and 0.162 FP/h. On the participant level, the algorithm had a mean sensitivity of 92.2% and 0.176 FP/h.

Number of subjects (*n*), Accelerometer (ACC), Electrodermal Activity (EDA), Electrocardiogram (ECG), Photoplethysmogram (PPG), Electroencephalogram (EEG), Blood Volume Pulse (BVP), Body Temperature (TEMP), Heart Rate (HR), Surface Electromyography (sEMG), Respiratory Inductance Plethysmography (RIP), Gyroscope (GYR), Random Forest (RF), k-Nearest Neighbor (kNN), Support Vector Machine (SVM), Neural Network (NN), Generative Adversarial Network (GAN), Gradient Tree Boosting Machine (GTBM), Long Short-term Memory (LSTM), Machine Learning (ML), Convolutional Neural Network (CNN).

### 4.1 Wristbands

Wrist-worn devices are the most widely studied wearables for seizure detection, as they can noninvasively measure heart rate, movement, and other physiological signals. The most frequently investigated model in the literature has been the Empatica E4 (Empatica Inc., Milan, Italy). Designed specifically for research, this wristband integrates four sensors to measure PPG, acceleration, body temperature, and EDA (28). These biosignals have been observed to fluctuate during several seizure types and have made the E4 a popular choice. While the E4 model is no longer available for purchase, it was previously priced at 1,690 USD (29), making it more suitable for research than large-scale consumer use. Empatica Inc. currently offers the EpiMonitor wristband, a United States Food and Drug Administration (FDA)-cleared medical device priced at 399 USD (30). The NightWatch armband sensor is a separate wearable that is CE-marked and recently received FDA clearance (31–33). It integrates PPG and acceleration to detect nocturnal motor seizures. The CE-marked Epi-Care free and Epi-Care mobile wristband sensors detect motor seizures using acceleration data, with the Epi-Care mobile system additionally alerting caregivers via a paired smartphone (34, 35). At present, both devices are marketed in Europe and have not received FDA authorization. Other wrist-worn devices that have been evaluated for seizure detection include the Apple Watch (Apple, CA, United States), Fitbit Charge 2 (Fitbit, CA, United States), Microsoft Band (Microsoft, WA, USA), Samsung SM-R800 Watch (Samsung Electronics Co., Ltd., South Korea), and additional prototypes (36–41). These devices differ in design and purpose, including epilepsy-specific monitors, fitness trackers, and consumer smartwatches. As costs decrease and accessibility expands, further research and development in wrist-worn detection systems are expected to accelerate.

### 4.2 Earpieces

Devices worn in- or over-the-ear have emerged as promising wearables for seizure detection. Unlike wristbands, which rely on indirect physiological measures, these devices can record EEG waveforms directly. An early study employing in-ear EEG placed four electrodes in each ear: one electrode in each of the cymba and the cavum of the concha, and two in the external auditory canal (42). EEG signals captured within the ear were shown to be comparable to signals captured from the scalp for seizure detection. Building on this work, a subsequent study simplified the design, placing two electrodes in each ear: one in each of the cymba and the cavum of the concha (43). Analysis found that the sensitivity of seizure detection from in-ear EEG recordings was comparable to scalp EEG and intracranial EEG. A related approach, behind-the-ear EEG, involves two electrodes glued to the skin behind each ear to capture electrical activity from the temporal lobes (44–48). Both in-ear and behind-the-ear approaches are limited anatomically by placement of temporal lobe electrodes only, which can result in missed seizure activity. Nevertheless, the compact design of these devices highlights their potential for everyday wearable use. While the FDA has not authorized any in-ear or over-the-ear EEG devices for seizure detection, mjn-SERAS is an in-ear device for seizure prediction that has received CE-marking in Europe (49).

### 4.3 Other emerging devices

Researchers have explored innovative systems that extend beyond traditional designs for medical devices. The Hexoskin connected shirt (Carré Technologies Inc., Canada) embeds textile sensors to measure physiological signals including ECG, respiratory inductance plethysmography, and acceleration (50). It enables long-term monitoring in a lightweight and less obtrusive form than hospital-based methods. However, challenges remain in improving signal clarity during daily activities and in validating performance during seizure events. These limitations, which also apply to wristbands and earpieces, highlight the need for advanced analytical approaches to extract meaningful information from the large volumes of data. In parallel, emerging devices are shifting from seizure detection toward seizure prediction, with the goal of identifying physiological changes preceding seizure onset and enabling seizure prevention strategies as we discuss more below (51–53).

## 5 Artificial intelligence

### 5.1 Machine learning methods

Machine learning (ML) encompasses the techniques of a computer identifying patterns in data, with many of these methods having been applied to seizure detection (54). Deep learning uses artificial neural networks, whereby the data are processed from an input layer through multiple hidden layers to an output layer (for example, the input layer could be EEG waveform data and the output layer could be the classification for the presence or absence of epileptiform abnormalities); the weights for the links between layers are adjusted during training in a method that is considered analogous to the strength of synapses between neurons (55). Support vector machines involve determining a hyperplane that facilitates the classification of data into different categories such as seizure or non-seizure state (56). Random forests utilize decision trees to determine classifications and are particularly useful for multimodal biosignals (57, 58). K-nearest neighbors classify data based on similarity to neighboring points (59). These techniques can also be used for regression, where a common output for a seizure classification device is a continuous number between 0 and 1 that is often referred to as a confidence score (i.e., scores closer to 1 indicate a greater confidence of seizure). More recent works have explored generative adversarial networks and gradient tree boosting machines, which augment limited datasets by generating artificial seizure-like signals and can improve performance (60, 61). While these models vary in complexity, their effectiveness is ultimately reflected in the performance achieved when applied to signals from wearable devices.

### 5.2 Data processing and reference standards

After signals are recorded by wearable devices, the resulting data streams must be organized and processed for further evaluation, which can be done manually (including by experienced epileptologists), automatically (including through ML algorithms), or a combination. Most studies rely on EEG review by a trained epileptologist as a reference standard to determine seizure onset and offset. In several studies, epileptologists visually evaluated the data after signals were scaled and transformed to ensure blinding to the EEG modality—intracranial, scalp, video, or ear. Additional preprocessing, including correcting for time drift or misalignments between devices, were often required. In studies employing ML, models were typically trained on annotated datasets and subsequently evaluated on independent test datasets to assess generalizability.

### 5.3 Performance

The performance of ML models in seizure detection has been evaluated across a range of devices and is typically assessed using sensitivity, the proportion of true seizure events that are correctly identified, and false alarm or false positive rate, commonly reported as false alarms per 24 h (FA/24 h). There is inherently a trade-off between maximizing sensitivity to capture true seizures and minimizing false positive alerts; it is also important to recognize that a single false alarm occurring every 24 h would likely be impractical for everyday use. Studies employing the Empatica E4 wristband provide useful benchmarks. Using a gradient tree boosting machine model applied to accelerometry and EDA data from the Empatica E4, Böttcher et al. reported a detection sensitivity of 91% with no false positive outputs in the test dataset, and 0.37 FA/24 h in an expanded test set (62). In a separate investigation with the same device, Nasseri et al. applied a recurrent neural network to accelerometry, blood volume pulse, EDA, body temperature and heart rate data, achieving a sensitivity of 93% with 2.3 FA/24 h for motor seizures and a sensitivity of 47% with 7.2 FA/24 h for all seizure types (63). Comparable sensitivity and false alarm rates have been reported in prototype wrist-worn devices. Larsen et al. evaluated acceleration and gyrometer signals using an artificial neural network (41). On the test dataset, the model achieved a sensitivity of 100% with 0.552 FA/24 h.

Parallel research has examined the potential of ML models for behind-the-ear EEG devices. Using the NicoletOne EEG system, which employs two electrodes behind each ear, You et al. constructed a generative adversarial network and achieved a sensitivity of 96.3% with 3.36 FA/24 h (45). In another study, Swinnen et al. used two Ambu Neuroline cup electrodes behind each ear to compare the performance of a support vector machine algorithm to the performance of experienced epileptologists (46). Human analysis found a median sensitivity of 81%, while the support vector machine algorithm had a sensitivity of 99.67% with 57.4 FA/24 h. Modifications to the initial algorithm reduced false positives to 21.9 FA/24 h with a slight decrease in sensitivity to 98.3%. More recently, Lehnen et al. evaluated the BrainSD device, which uses two silicone earpieces each equipped with two electrodes (48). Across multiple algorithms, support vector machines performed best with 95.3% accuracy and 97% sensitivity; k-nearest neighbors and random forest models both achieved 92.8% accuracy. Further validation in large-scale studies is necessary to determine the feasibility of ear-EEG as an option for long-term seizure monitoring in the outpatient setting.

### 5.4 Regulations

Regulatory considerations impact the potential clinical use of wearable medical devices that incorporate AI. In the United States, the FDA regulates AI-enabled software used for diagnosis or prevention of disease as medical devices (64). The regulatory process allows consideration of the benefits and risks of each device and will commonly involve a validation study. When incorporated into wearable devices, many algorithms are ‘fixed-and-frozen’ such that software modifications cannot be made without further regulatory review. Additionally, the data generated by these devices may be subject to the Health Insurance Portability and Accountability Act (HIPAA), which establishes strict standards for the protection of health-related data. Manufacturers of seizure-detection technologies must address device safety and data privacy in parallel.

## 6 Future outlook

Recent studies introduce ML models for seizure detection. Improving signal-to-noise ratios and developing detection algorithms that enhance sensitivity without amplifying false alarm rates persist as the main challenges in optimizing performance. There is an inherent positive feedback loop with the adoption of wearable technology: increased use can provide more training data to hopefully improve performance and drive further adoption.

Concurrently, research groups are investigating algorithms that can predict seizures using wearable technologies. This ability to forecast seizures aims to enable people living with epilepsy to plan accordingly for a seizure, including ensuring they are in a safe location and implementing preventative strategies. In a survey of people living with epilepsy and their caregivers, 76% indicated that they would use a device that could predict times of high seizure chance and low seizure chance (65). Similar to detection algorithms, predictive algorithms require a balance between acceptable sensitivity and false alarm rate, with the added challenge of providing predictions with sufficient time to allow intervention. One study aimed to predict a seizure occurring in people admitted to an epilepsy monitoring unit over the coming day (53). It used a 15-min recording with the Empatica E4 wristband alongside clinical data and achieved an average classification of 66%. The integration of multimodal physiological signals, which may also include broader wearable signals such as sleep phase duration, will likely play a key role in developing models with acceptable performance.

## 7 Conclusion

Wearables for seizure detection represent an important advancement for individuals living with epilepsy. Although current devices vary in accuracy, improvements in the integration of physiological signals and in algorithm performance demonstrate meaningful progress toward real-world application. For clinicians, these systems extend monitoring beyond the hospital setting, enabling timely interventions and personalized treatment. For scientists and engineers, the next frontiers lie in identifying which biosignals most reliably predict seizures and in designing algorithms that achieve consistent performance across diverse populations. Ultimately, the goal is to develop a device that is accurate, reliable and affordable without impeding daily life. Such a device would empower individuals to monitor their own health while keeping caregivers and clinicians connected—bringing together technological innovation and improved neurological outcomes.
